# Common variation at 16p11.2 is associated with glycosuria in pregnancy: findings from a genome-wide association study in European women

**DOI:** 10.1093/hmg/ddaa054

**Published:** 2020-03-30

**Authors:** Matthew A Lee, George McMahon, Ville Karhunen, Kaitlin H Wade, Laura J Corbin, David A Hughes, George Davey Smith, Debbie A Lawlor, Marjo-Riitta Jarvelin, Nicholas J Timpson

**Affiliations:** 1 MRC Integrative Epidemiology Unit at University of Bristol, Bristol BS8 2BN, UK; 2 Population Health Sciences, Bristol Medical School, University of Bristol, Bristol BS8 2BN, UK; 3 Faculty of Medicine, School of Public Health, Imperial College London, 156 Norfolk Place, St Mary’s Campus, London W2 1PG, UK; 4 Faculty of Medicine, Northern Finland Birth Cohort Studies and Center for Life Course Health Research, University of Oulu, Aapistie 5 B, Oulu Fin-902200, Finland; 5 Medical Research Council Integrative Epidemiology Unit, Avon Longitudinal Study of Parents and Children, Population Health Science, Bristol Medical School, Oakfield House, Oakfield Grove, Bristol BS8 2BN, UK

## Abstract

Glycosuria is a condition where glucose is detected in urine at higher concentrations than normal (i.e. not detectable). Glycosuria at some point during pregnancy has an estimated prevalence of 50% and is associated with adverse outcomes in both mothers and offspring. Little is currently known about the genetic contribution to this trait or the extent to which it overlaps with other seemingly related traits, e.g. diabetes. We performed a genome-wide association study (GWAS) for self-reported glycosuria in pregnant mothers from the Avon Longitudinal Study of Parents and Children (cases/controls = 1249/5140). We identified two loci, one of which (lead SNP = rs13337037; chromosome 16; odds ratio of glycosuria per effect allele: 1.42; 95% CI: 1.30, 1.56; *P* = 1.97 × 10^**−13**^) was then validated using an obstetric measure of glycosuria measured in the same cohort (227/6639). We performed a secondary GWAS in the 1986 Northern Finland Birth Cohort (NFBC1986; 747/2991) using midwife-reported glycosuria and offspring genotype as a proxy for maternal genotype. The combined results revealed evidence for a consistent effect on glycosuria at the chromosome 16 locus. In follow-up analyses, we saw little evidence of shared genetic underpinnings with the exception of urinary albumin-to-creatinine ratio (*R*_**g**_ = 0.64; SE = 0.22; *P* = 0.0042), a biomarker of kidney disease. In conclusion, we identified a genetic association with self-reported glycosuria during pregnancy, with the lead SNP located 15kB upstream of *SLC5A2,* a target of antidiabetic drugs. The lack of strong genetic correlation with seemingly related traits such as type 2 diabetes suggests different genetic risk factors exist for glycosuria during pregnancy.

## Introduction

At normal levels, glucose is not detectable in urine. Glycosuria, the presence of glucose in urine above normal levels, may be caused by either an increase in blood glucose such that the renal tubules are overwhelmed and complete reabsorption of presented glucose is not possible, a lowering of the renal threshold, or inhibition of renal tubule reabsorption (1). Although prevalence estimates for glycosuria vary, it seems likely that the condition effects around 50% of women at some stage of their pregnancy (2) and is thought to occur primarily as a result of pregnancy-related increases in renal blood flow resulting in a lower threshold for excreting glucose in urine. It also reflects pregnancy-related increases in circulating blood glucose. While, outside of pregnancy, glycosuria is considered a diabetes indicator, glycosuria in pregnancy is not specific or sensitive for gestational diabetes risk and is not recommended as a screening tool for it (2,3).

There is evidence that glycosuria is associated with adverse cardio-metabolic outcomes, including non-alcoholic fatty liver disease, in offspring (4–6) and with later life outcomes, such as cardiovascular disease death, in mothers (7). Therefore, the presence of glycosuria may potentially indicate future adverse outcomes in pregnancy and the life course.

Both glucose regulation and renal function are heritable, with reliable evidence for several genetic variants being associated with a range of relevant traits, including fasting glucose (8,9), insulin sensitivity (8,9), type 2 diabetes (10) and glomerular filtration rate in general non-pregnant populations (11). While these studies have yielded valuable new insights into glucose metabolism, still outstanding is an investigation of the genetic contribution to glycosuria during pregnancy. To investigate whether common genetic variants are associated with glucose in urine during pregnancy and to explore potential links to other glycaemic and renal-function-related traits, we set out to conduct a genome-wide association study (GWAS) of glycosuria during pregnancy in the Avon Longitudinal Study of Parents and Children (ALSPAC) and looked for confirmatory evidence in the Northern Finland Birth Cohort 1986 (NFBC1986). We further investigated the genetic overlap between identified loci and multiple diabetes-related traits.

## Results

**Figure 1:**
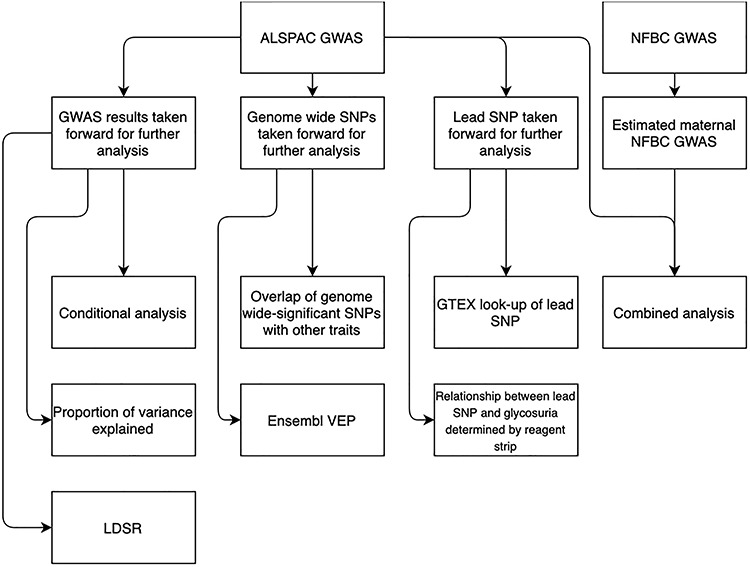
Analysis overview. The flowchart provides an overview of GWAS follow-up analysis.

A genome-wide association study (GWAS) of self-reported glycosuria during pregnancy performed in ALSPAC represents our primary analysis. Additional work exploring associations further was conducted in the same cohort. Due to lack of available data, a replication GWAS was not possible. We instead sought supporting evidence from a secondary GWAS analysis performed in NFBC1986 and combined GWAS results from ALSPAC and NFBC1986 to identify persistent associations. An overview of the study and its components is presented in Figure 1 and Supplementary Material, Figure S1.

## Primary Analysis

### ALSPAC GWAS

**Figure 2:**
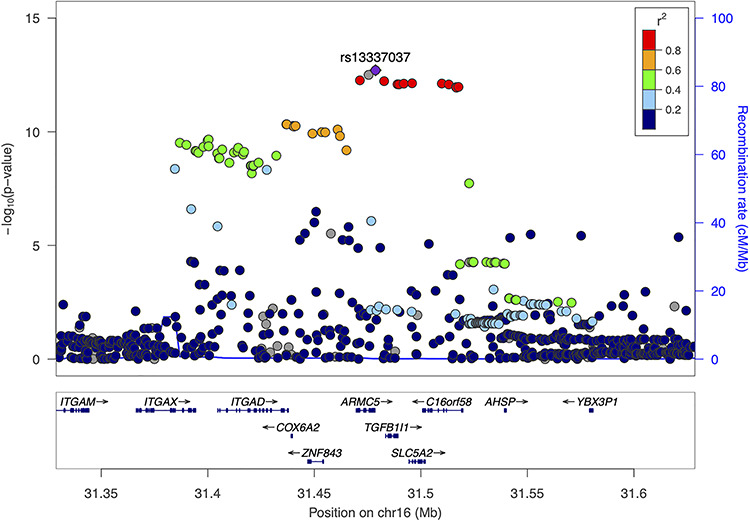
Regional association plot of lead association on chromosome 16 (rs13337037) in ALSPAC GWAS. *P*-values (on a −log10 scale) in ALSPAC are shown. Each SNP is coloured according to the degree of LD with the lead SNP rs13337037 (shown as a purple diamond). LD values are from the 1000 Genomes (March 2012 release) European population (created using locuszoom.org build: hg19).

Assuming an additive genetic model, the estimated variance explained by genotype across all variants in ALSPAC was estimated to be 1.6% (standard error (SE) = 0.08), corresponding to an estimated narrow-sense heritability of 10% (SE = 0.05, 12–14). There was evidence for association at genome-wide significance (*P*-value = 5 × 10^−8^) at 55 SNPs (Supplementary Material, Figures S2, S3 and Supplementary Material, Table S1) across chromosomes 16 (Fig. 2) and 9 (Supplementary Material, Figure S4). The lead SNP on chromosome 16 was rs13337037 (odds ratio (OR) of glycosuria per A allele: 1.42; 95% confidence interval (CI): 1.30, 1.56; *P*-value = 1.97 × 10^−13^; minor allele frequency (MAF) = 28%; Fig. 2) and the only SNP on chromosome 9 was rs10991823 (OR of glycosuria per T allele: 1.47; 95% CI: 1.29, 1.68; *P*-value = 4.02 × 10^−8^; MAF = 10%; Supplementary Material, Figure S4). The lead SNP on chromosome 16 is part of a block of 17 SNPs in high linkage disequilibrium (LD; *R*^2^ > 0.8; Fig. 2) in a region containing multiple genes: *ARMC5, TGFB1I1, SLC5A2* and *C16orf58.* The lead SNP on chromosome 9 is part of a block of 25 SNPs in high LD (*R*^2^ > 0.8; Supplementary Material, Figure S4) surrounding the *AUH* gene. Conditional analysis did not reveal any additional associations on either chromosome.

The correlation between self-reported glycosuria and glycosuria determined by reagent strip was 0.31 (*P*-value = 2.2 × 10^−16^; Supplementary Material, Table S2). Logistic regression showed the association of rs13337037 with glycosuria was stronger (with overlapping confidence intervals) when glycosuria was determined by reagent strip (OR per A allele of a positive reagent strip test result: 1.64; 95% CI: 1.35, 2.00; *P*-value = 6.72 × 10^−7^). The corresponding OR for rs10991823 was 1.10 (OR per T allele of a positive reagent strip test result: 1.10; 95% CI: 0.82, 1.48; *P*-value = 0.51).

### Secondary analysis

In a GWAS performed in NFBC1986 using offspring genotype and maternal midwife-reported glycosuria, there was evidence for an association (*P*-value = 5 × 10^−8^) at 49 SNPs on chromosome 6 (Supplementary Material, Figures S5, S6 and Supplementary Material, Table S3). To evaluate the persistence of signal across the two cohorts, association results were combined. This revealed evidence for a consistent effect on glycosuria at rs13337037 on chromosome 16 only (Z-score: 7.833; *P*-value: 4.75 × 10^−15^; effect allele = A; MAF = 27%) (Supplementary Material, Figures S7, S8 and Supplementary Material, Table S4). The effect estimate for rs13337037 in NFBC1986 was OR: 1.25 (5% CI: 1.12, 1.38; *P*-value = 9.8 × 10^−4^; effect allele = A; MAF = 26%). The re-scaling of this effect size to approximate the allelic effect in mothers (15,16), as in ALSPAC, gave an OR of 1.57 (95% CI, 1.30, 1.83). Results of analyses undertaken to test the validity of this re-scaling are presented in Supplementary Information and Supplementary Material, Table S5.

### Additional analysis

#### Overlap of genome-wide significant SNPs in ALSPAC with other GWASs

None of the SNPs reaching genome-wide significance (*P*-value = 5 × 10^−8^) in the primary analysis showed evidence of association with fasting insulin (8), fasting glucose (8), HbA1c (9), type 2 diabetes (10), BMI (17) or estimated glomerular filtration rate (11) at a genome-wide level of significance (Supplementary Material, Table S5). In a comparison of effect estimates of SNPs associated with each of these traits and the estimates for the same SNPs in our GWAS of glycosuria, the strongest correlation was found with estimated glomerular filtration rate (*r*^2^ = −0.44; 95% CI = −0.54, −0.34; *P*-value = 5.78 × 10^−13^), with weaker correlations found for type 2 diabetes (*r*^2^ = 0.08; 95% CI = 0.04, 0.13; *P*-value = 0.0005) and HbA1c (*r*^2^ = −0.07; 95% CI = −0.14, −0.001; *P*-value = 0.046) (Supplementary Material, Figure S9).

#### Linkage disequilibrium score regression

Of the 832 traits available in LD Hub (date accessed: 19/02/2019), genetic correlations were not returned for 81 traits, with a further two traits showing correlations above 1 and below −1 (LDSR mean Chi^2^ = 1.022). Our main focus was on trait categories from LD Hub that were a priori related to glycosuria (anthropometric, cardiometabolic, glycaemic, kidney, reproductive), and results for these are shown in Supplementary Material, Table S6. Of the 43 traits from the categories of interest, those that showed the strongest genetic correlation with our glycosuria trait were urinary albumin-to-creatinine ratio in non-diabetes (*R*_g_ = 0.7; SE = 0.24; *P*-value = 0.004) and urinary albumin-to-creatinine ratio (*R*_g_ = 0.64; SE = 0.22; *P*-value = 0.004).

#### Functional informatics

We passed the rs13337037-defined signal (including SNPs in high LD) through the Ensembl Variant Effect Predictor. All SNPs were existing variants with the ‘MODIFIER’ impact annotation, suggesting they are non-coding variants or variants affecting non-coding genes (Supplementary Material, Table S7). rs13337037 is a non-coding variant downstream of *ARMC5* (a member of the ARM (armadillo/beta-catenin-like repeat) superfamily implicated in mediation of protein–protein interactions (18)) and upstream of *TGFB1I1* (coactivator of the androgen receptor (19)) and *SLC5A2*; there was a weak evidence of an impact on any of these genes from the lead SNP (Supplementary Material, Table S7).

In GTEx, there was evidence that rs13337037 acts as a cis-eQTL (expression quantitative trait locus) for a number of nearby genes, including *ARMC5, TGFB1I1, SLC5A2* and *ZNF843* across a number of different tissues (Supplementary Material, Table S8).

## Discussion

In a GWAS analysis of glycosuria during pregnancy conducted in ALSPAC (a European longitudinal cohort), we identified an association between a block of common SNPs with high LD surrounding multiple genes (*ARMC5, TGFB1I1, SLC5A2* and *C16orf58*) on chromosome 16 and self-reported phenotype. When examining the relationship between rs13337037 and glycosuria determined by reagent strip, we found consistent evidence for association despite differences in sample size and the frequency of glycosuria status. Each A allele of rs13337037 was associated with a 47% increased risk of self-reported glycosuria and a 64% increased risk of glycosuria determined by reagent strip. While we lacked fully replicable datasets for our GWAS of self-reported glycosuria, we instead made use of offspring genotype as a predictor for maternal genotype in the NFBC1986 collection. The only signal that persisted in efforts to filter out chance associations in ALSPAC by combining GWAS results from the two cohorts was that at rs13337037 on chromosome 16.

rs13337037 lies 15 kb upstream of *SLC5A2* and, while it is difficult to assert a direct relationship between neighbouring loci and signal at specific SNPs (20), evidence has implicated *SLC5A2* in familial renal glycosuria (21,22). *SLC5A2* encodes a low-affinity, high-capacity Na(+)/glucose cotransporter (SGLT2, sodium glucose cotransporter 2) (23). For the other genes surrounding the lead SNP on chromosome 16, their relation to glycosuria is unclear: *ARMC5* is implicated in mediation of protein–protein interactions (18) and may act as a tumour suppressor (24) (date accessed: 02/22/2019—https://www.ncbi.nlm.nih.gov/gene/79798); *TGFB1I1* encodes a protein that acts as a coactivator of the androgen receptor (19) and which may be involved in prostate cancer (25) (date accessed: 02/22/2019—https://www.ncbi.nlm.nih.gov/gene/7041); *C16orf58* is not well characterized but is thought to be involved in protein–protein interaction (date accessed: 02/22/2019—https://www.ncbi.nlm.nih.gov/gene/64755).

Sodium glucose cotransporters mediate the reabsorption of glucose in the kidney, with the *SLC5A2-*encoded SGLT2 responsible for 90% of this glucose reabsorption and SGLT1 (high affinity and low capacity) responsible for the remaining ~ 10% (23). Studies show SGLT2 levels are increased in diabetic patients compared with non-diabetics, though the mechanism for this is not understood (1). In adults with type 2 diabetes, pharmaceutical targeting of renal glucose reabsorption by inhibition of SGLT2 can be successful at improving glycaemic control via excretion of glucose in urine (26). These gliflozin inhibitors act independently of insulin, providing a novel therapeutic avenue (26). Aberrant expression of *SGLT2* is known to result in glycosuria (23), though, due to mutations within the *SLC5A2* locus mostly being within families, common mutations have yet to be identified. In GTEx, there is evidence that rs13337037 acts as a cis-eQTL for *SLC5A2*.

In a clinical setting, the relationship between glycosuria during pregnancy and other related metabolic traits such as fasting blood glucose and type 2 diabetes (either during pregnancy or more generally) is unclear. In our exploration of the shared genetic contribution to glycosuria during pregnancy and other metabolic traits, we found that while there was no overlap in genome-wide significant hits across the traits, estimates of shared heritable contribution (i.e. considering genetic effects across the entire genome) identified urinary albumin-to-creatinine ratio as the most strongly correlated trait. While the LDSR approach used for these analyses provides only an approximation of genetic correlation and results should ideally be followed up by more detailed analyses (27), these preliminary findings suggest that renal function may be an important component of glycosuria.

### Limitations

We used a glycosuria measurement that was retrospectively reported by mothers in pregnancy for our main ALSPAC analyses. This is likely to be reported with error and will likely include women with very different levels of glycosuria (for example, it may include women who were told they had ‘a trace of sugar’ in their urine as well as those with higher levels). This is supported by the higher prevalence of glycosuria defined by retrospective report compared with the definition used for glycosuria determined by reagent strip of at least ‘++’ on at least two occasions from all glycosuria measures in antenatal records (20% versus 4%). However, these two measures were correlated. Any misclassification of this maternal retrospective report is likely to be non-differential by genotype (as the women will not know their genotype) and so would be expected to attenuate any genome-wide results towards the null. This is consistent with our finding that the lead SNP was more strongly associated with the reagent strip measure of glycosuria than with the retrospective report.

In the absence of other studies with both glycosuria assessed during pregnancy and corresponding associated genome-wide genetic data, we were limited in our ability to perform an independent replication of the GWAS performed in ALSPAC. However, we were able to look for persistent signal using results from a GWAS performed in NFBC1986 using offspring genotype. It has been suggested that allelic effect estimates derived in this way can be re-scaled (multiplied by 2) to give an approximation of the allele effect in the mother. However, given that this re-scaling is based on expectation, one can expect the actual multiplication factor, in any given instance, to vary around this expectation. Furthermore, we did not carry out a comprehensive validation of this approach within the context of this study, where additional issues such as the potential for a direct effect of offspring genotype on maternal glycosuria phenotype could be relevant. Therefore, while the combined results indicate persistence of the primary signal across the two datasets and we see reasonable agreement with respect to the magnitude of effect in the two studies, our results should be treated with caution until replicated, preferably in independent large cohorts where both maternal genotype and phenotype are available.

### Summary

Our GWAS of reported glycosuria during the third trimester of pregnancy has identified associations of genetic variants on chromosome 16 near multiple genes, including *SLC5A2*, which has been implicated in familial renal glycosuria and the gene product of which is the target of some type 2 diabetes drugs (gliflozins, e.g. Dapagliflozin). Furthermore, hypothesis-free genetic correlation analysis suggested genetic loci underpinning glycosuria susceptibility may be linked with kidney disease, reflecting the joint role of circulating glucose and renal function on glycosuria and the possible effect of hyperglycaemia on renal function. This shared heritability suggests distinguishing gestational diabetes from other presentations of diabetes or kidney disease may not be possible. Further replication of these findings is important.

## Materials and Methods

### ALSPAC overview

Participants were mothers from ALSPAC, a large prospective cohort study that recruited 14 541 pregnancies in the former Avon Health Authority area in South West England, with expected delivery dates between the 1^st^ April 1991 and the 31^st^ December 1992 (28,29) (see Supplementary Information, ALSPAC Overview, for full details). The study website (http://www.bristol.ac.uk/alspac/) contains details of all the data that is available through a fully searchable data dictionary and variable search tool (http://www.bristol.ac.uk/alspac/researchers/our-data/). Ethical approval for the study was obtained from the ALSPAC Ethics and Law Committee and the Local Research Ethics Committees (http://www.bristol.ac.uk/alspac/researchers/research-ethics/). Informed consent for the use of data collected via questionnaire and clinics was obtained from participants following recommendations of the ALSPAC Ethics and Law Committee at the time. Full details of the ALSPAC consent procedures are available on the study website (http://www.bristol.ac.uk/alspac/researchers/research-ethics/).

### ALSPAC phenotype data

#### Self-reported glycosuria

We used two measures of glycosuria in ALSPAC (Supplementary Material, Figure S1). Firstly, mothers were sent a pregnancy-related self-report questionnaire approximately 8 weeks after the end of their pregnancy, which included the following question: ‘During the last months of pregnancy (from seven months onwards) did you experience sugar in urine?’, with possible answers ‘yes, in the last months of pregnancy’, ‘no, not in the last months of pregnancy’ and ‘don’t know’. In total, 11 710 women returned the questionnaire and 11 660 (99%) answered this question, with 2389 (21%) responding ‘yes, in the last months of pregnancy’. Of the 11 660 women who answered the glycosuria question, 7429 (64%) had genetic data and, of these, 1519 (20%) self-reported having glycosuria.

At recruitment, women were also asked about existing diabetes and any previous history of gestational diabetes. Women were classified into one of four mutually exclusive categories: no evidence of glycosuria or diabetes (*n* = 11 773), existing diabetes before the pregnancy (*n* = 47), gestational diabetes (i.e. a diagnosis written in the medical records of gestational diabetes in any woman with no history of existing diabetes; *n* = 57) and glycosuria (i.e. ++ glycosuria on two occasions in women with no evidence of existing or gestational diabetes; *n* = 404) (4,5). Of the 7429 women with phenotype and genetic data, we excluded those without information on pre-existing diabetes and gestational diabetes and glycosuria, and those with pre-existing diabetes or gestational diabetes, leaving 7089 (61% of 11 660 respondents), of whom, 1399 (20%) self-reported having glycosuria. After excluding women who had withdrawn consent for the phenotype and genetic data, and related individuals, 6639 (93%) women had self-reported glycosuria data and genotype data. Finally, we removed women who did not have information on our second measure of glycosuria (glycosuria determined by reagent strip), leaving 6389 (96%; 55% of 11 660) women of whom 1249 (20%) self-reported having glycosuria.

#### Reagent strip defined glycosuria

Due to potential misreporting in the postnatal questionnaire response, we also generated a variable based on objectively measured reagent strip tests of pregnancy glycosuria (glycosuria determined by reagent strip). Information on glycosuria (recorded in the obstetric records as none, trace, +, ++, +++ or more) was abstracted from the records of each antenatal clinic visit made by the woman (median number, 12 per woman (interquartile range: 9–14)). Glycosuria was defined as a record of at least ++ (equal to 13.9 mmol/l or 250 mg/100 mL) on at least two occasions at any time during the pregnancy. In total, 12 281 women had information on reagent strip defined glycosuria.

Of the 12 281 women with information on reagent-strip-determined glycosuria, we excluded those without information on pre-existing diabetes and gestational diabetes and glycosuria, and those with pre-existing diabetes or gestational diabetes. Of the remaining 12 177 women, 404 (3%) fulfilled our criteria for glycosuria and 7652 (63%) had available genetic data. Of the 7652 women, 261 (3%) fulfilled our criteria for glycosuria. After excluding women withdrawing consent for the phenotype and genetic data, as well as related individuals, 6866 (91%) women had data on glycosuria determined by reagent strip and genotype data, of which 227 (3%) fulfilled our criteria for glycosuria.

### ALSPAC genotype data

Imputation of ALSPAC mother’s genetic data was performed on a combined mother and child data set using Impute2 against the 1000 Genomes Phase 1 reference panel (30). ALSPAC mothers were genotyped using the Illumina human660W-quad array at Centre National de Génotypage (CNG). For ALSPAC mothers, SNPs with a minor allele frequency (MAF) of <1%, a call rate of <95%, or evidence for violations of Hardy–Weinberg equilibrium (*P* < 1 × 10^−06^) were removed. ALSPAC children were genotyped using the Illumina HumanHap550 quad chip genotyping platforms by 23andMe subcontracting the Wellcome Trust Sanger Institute, Cambridge, UK and the Laboratory Corporation of America, Burlington, NC, USA. For ALSPAC children, SNPs with MAF of <1%, a call rate of <95% or evidence for violations of Hardy–Weinberg equilibrium (*P* < 5 × 10^−7^), were removed. In total, we removed 1946 individuals based on relatedness and withdrawal of consent for both genotype and the phenotype data, leaving 15 896 individuals for further analysis, of which 7921 were mothers. See Supplementary Information for full details on ALSPAC genetic data and imputation.

### NFBC1986 overview

The Northern Finland Birth Cohort 1986 (NFBC1986) is a prospective birth cohort that recruited women in the two northernmost provinces of Finland (Oulu and Lapland) with an expected delivery date between 1^st^ July 1985 and 30^th^ June 1986. A total of 9362 mothers were included in the study, resulting in 99% of all eligible births in the region being recruited (*n* = 9432 live born children) (31)(Supplementary Material, Figure S1). Informed written consent was obtained from all participants, and the research protocols were approved by the Ethics Committee of Northern OStrobothnia Hospital District, Finland.

### NFBC1986 phenotype data

Data were gathered prospectively at the first antenatal clinic visit and onwards (mean gestational age at inclusion = 12 weeks). Mothers provided information on maternal background via self-report questionnaires. Data on antenatal visits, hospital admissions and birth outcomes were obtained from maternity health centres and hospital medical records. Midwives were given questionnaires to complete during the mother’s last visit to the antenatal care unit or during the first home visit after the delivery, which included the following question: ‘urinary glucose’ with possible answers ‘+’ (equivalent to yes) and ‘−’ (equivalent to no) (31). Pregnancy questionnaires are available at the NFBC1986 pregnancy and antenatal data website (https://www.oulu.fi/nfbc/node/18143), with information on glycosuria reported in ‘Pregnancy Questionnaire II (yellow form)’, question 7.

Of the 9362 women included in the study, 9336 had data available on midwife-reported glycosuria. Of these 9336, 1789 (19%) were reported with ‘+’. Of the 9336 women with available midwife-reported glycosuria, 3738 (40%) had quality-controlled offspring genetic data available and, of these mothers, 747 (20%) were reported as having glycosuria.

For this GWAS, maternal genotype data were not available and so we tested for an association of offspring genotype with midwife-reported glycosuria as our main outcome.

### NFBC1986 genotype data

In the absence of genetic data for the mothers in NFBC1986, we used offspring genome-wide data, as a proxy for maternal genotype in this cohort. NFBC1986 children were invited to clinic at age 16 and 3834 individuals with consent were genotyped using the Illumina OmniExpressExome-8v1.2 Chip and the GenomeStudio algorithm. Quality control comprised of exclusions based on sex mismatch, outlying heterozygosity, duplicate samples and relatedness, after which genotype data were available for 3743 individuals—five of which had no information on mother’s glycosuria during pregnancy. After excluding SNPs not in Hardy–Weinberg equilibrium (*P* < 0.0001) or with low call rate (<0.99), imputation was performed using Impute2 against the 1000 Genomes Phase 3 reference panel (30). The total number of individuals with genotype and phenotype information was 3738 and of these 747 (20%) had glycosuria.

### Primary analysis

#### ALSPAC GWAS

The total number of variants after imputation equalled 28 699 509. Prior to genome-wide analysis, SNPs were filtered based on an info score threshold of ≥0.3 and a MAF threshold of ≥0.01, leaving 9 323 831 variants in the analysis. Testing an additive genetic model, we carried out a GWAS of imputed data on self-reported glycosuria in the third trimester of pregnancy (1249 cases and 5140 controls) using logistic regression and adjusting for the top 10 principal components of genetic ancestry to control for potential confounding by population stratification in SNPTEST (v2.5.2). We present all GWAS results and focus on those that satisfy a conventional genome-wide significance threshold of *P*-value ≤5 × 10^−8^. Full details of how the GWAS was performed, including how principal components were generated, are provided in the Supplementary Information. We estimated the proportion of variance explained by all SNPs in our GWAS using GCTA (v1.26.0) (12–14). We carried out conditional analysis with the lead SNPs coded as an additive effect for evidence of secondary signals on the same chromosome using SNPTEST (v2.5.2) and appraised the pattern of association at our lead SNPs using LocusZoom (32).

#### Within-cohort validation

We examined the relationship between self-reported glycosuria and glycosuria determined by reagent strip performing a Pearson’s correlation test in R (33) (version R-3.5). We investigated the relationship between glycosuria determined by reagent strip and the lead SNPs, assessing the odds per effect allele of a positive response for reagent strip glycosuria using logistic regression in R.

### Secondary analysis

#### NFBC1986 GWAS

The total number of variants after imputation equalled 81 571 831. We performed a logistic regression GWAS using SNPTEST (version 2.5) with the mother’s glycosuria in pregnancy as the outcome and offspring’s imputed genotype as the predictor of interest, assuming an additive model and including the top four multidimensional scaling (MDS) coordinates as covariates to adjust for population stratification (34). We filtered results based on an info score threshold of ≥0.3 and an MAF threshold of ≥0.01, resulting in 9 873 828 variants. We present all GWAS results and focus on those that satisfy a conventional genome-wide significance threshold of *P*-value ≤5 × 10^−8^.

Based on quantitative genetics theory (i.e. the rules of inheritance) and assuming an additive genetic effect, we expected that any allelic effect estimated in the NFBC1986 analysis (i.e. using offspring genotype in place of mother’s own) would be smaller compared to that observed using own genotype. More specifically, it has previously been suggested that the average (expected) allelic effect of one allele in the offspring (on the parental phenotype) is half the effect that would be observed using the parent’s own genotype (15,16). Further consideration of the validity of this re-scaling approach in the context of this study is presented in the Supplementary Information.

#### Combined analysis

In order to evaluate the persistence of signal across the two cohorts, association results from the two cohorts were combined. Given the different designs of the two analyses (i.e. the substitution of offspring genotype for mother’s genotype in the NFBC1986 GWAS), we performed a sample size-based approach in which the direction of effect and *P-*value observed in each study are converted into a signed Z-score indicating the strength and direction of effect (35). *Z*-scores for each allele were then combined across studies in a weighted sum, with weights proportional to the square root of the effective sample size for each study. We present all combined results and consider any associations reaching a conventional genome-wide significance threshold of *P*-value ≤5 × 10^−8^ as providing evidence of a persistent signal.

### Additional analysis

#### Overlap of genome-wide significant SNPs in ALSPAC with other GWASs

To investigate evidence of overlap of association between glycosuria and glucose regulation and renal function traits, we examined whether SNPs associated with glycosuria in pregnancy in ALSPAC are also associated with the following traits at a genome-wide significance threshold of *P*-value ≤5 × 10^−8^: HBA1c (9), fasting insulin (8), fasting glucose (8), type 2 diabetes (10), BMI (17), estimated glomerular filtration rate (11) (see Supplementary Information for full details of summary level data used). In addition, we explored concordance between effect estimates for SNPs reaching genome-wide significance (*P*-value ≤5 × 10^−8^) extracted from GWAS for the aforementioned traits and effect estimates for the same SNPs in our GWAS of glycosuria. Data were visualized in scatter plots and concordance assessed via Pearson’s product-moment correlation. The numbers of SNPs included in this analysis were as follows: type 2 diabetes = 1792, estimated glomerular filtration rate = 4196, BMI = 1860, fasting glucose = 290 and HBA1c = 821.

#### Linkage disequilibrium score regression

Using results from the ALSPAC GWAS, we estimated the genetic correlation of glycosuria with the 832 available traits in LD Hub (http://ldsc.broadinstitute.org/ldhub/) using linkage disequilibrium (LD) score regression (LDSR) (36).

#### Functional informatics

To identify potential functional roles of lead SNPs, we examined the effects of lead SNPs and SNPs in high LD (*R*^2^ ≥ 0.8) on genes and regulatory regions via the Ensembl Variant Effect Predictor (37). We examined the effect of lead SNPs in GTEx (20).

## Data and Material Availability

Data are available from the Northern Finland Birth Cohort (NFBC) for researchers who meet the criteria for accessing confidential data. Please contact NFBC project centre (NFBCprojectcenter@oulu.fi) and visit the cohort website (www.oulu.fi/nfbc) for more information.

Code for the analysis and visualization of the data is available at: https://github.com/mattlee821/001_glycosuria_GWAS

Summary statistics from GWAS are available at data.bris—https://doi.org/10.5523/bris.9vjsikubd658257lbu6lrizog

ALSPAC data used for this submission will be made available on request to the ALSPAC Executive via the website, which also provides full details and distributions of the ALSPAC study variables: http://www.bristol.ac.uk/alspac/researchers/access/. The ALSPAC data management plan (available here: http://www.bristol.ac.uk/alspac/researchers/data-access/documents/alspac-data-management-plan.pdf) describes in detail the policy regarding data sharing. A sample set of similar data containing relevant ALSPAC variables is available from the European Genome-phenome Archive (accession number: EGAS00001000090): https://www.ebi.ac.uk/ega/studies/EGAS00001000090.

## Author Contributions

Matthew A. Lee, George McMahon and Ville Karhunen are responsible for data curation, formal analysis, investigation, visualization, writing—original draft preparation. Dr Kaitlin H. Wade, Dr Laura J Corbin and Dr David A Hughes supervised and were responsible for writing—review and editing. Professor George Davey Smith and Professor Debbie A Lawlor were responsible for funding acquisition, writing—review and editing. Professor Marjo-Riitta Jarvelin for conceptualization, funding acquisition, project administration, supervision, writing—review and editing. Professor Nicholas J. Timpson for conceptualization, funding acquisition, methodology, project administration, supervision, writing—review and editing.

## Supplementary Material

Supplementary_Figure_1_ddaa054

Supplementary_Figure_2_ddaa054

Supplementary_Figure_3_ddaa054

Supplementary_Figure_4_ddaa054

Supplementary_Figure_5_ddaa054

Supplementary_Figure_6_ddaa054

Supplementary_Figure_7_ddaa054

Supplementary_Figure_8_ddaa054

Supplementary_Figure_9_ddaa054

Supplementary_tables_ddaa054

Supplementary_legends_ddaa054

HMG-2019-TF-00473_R1_Supplement_LEE_ddaa054
