# Supplementary figures and images for: Common variation at 16p11.2 is associated with glycosuria in pregnancy: findings from a genome-wide association study in European women
Source: Hum Mol Genet. 2020 Mar 30;29(12):2098–106. doi: 10.1093/hmg/ddaa054 (PMC7390941; doi:10.1093/hmg/ddaa054)

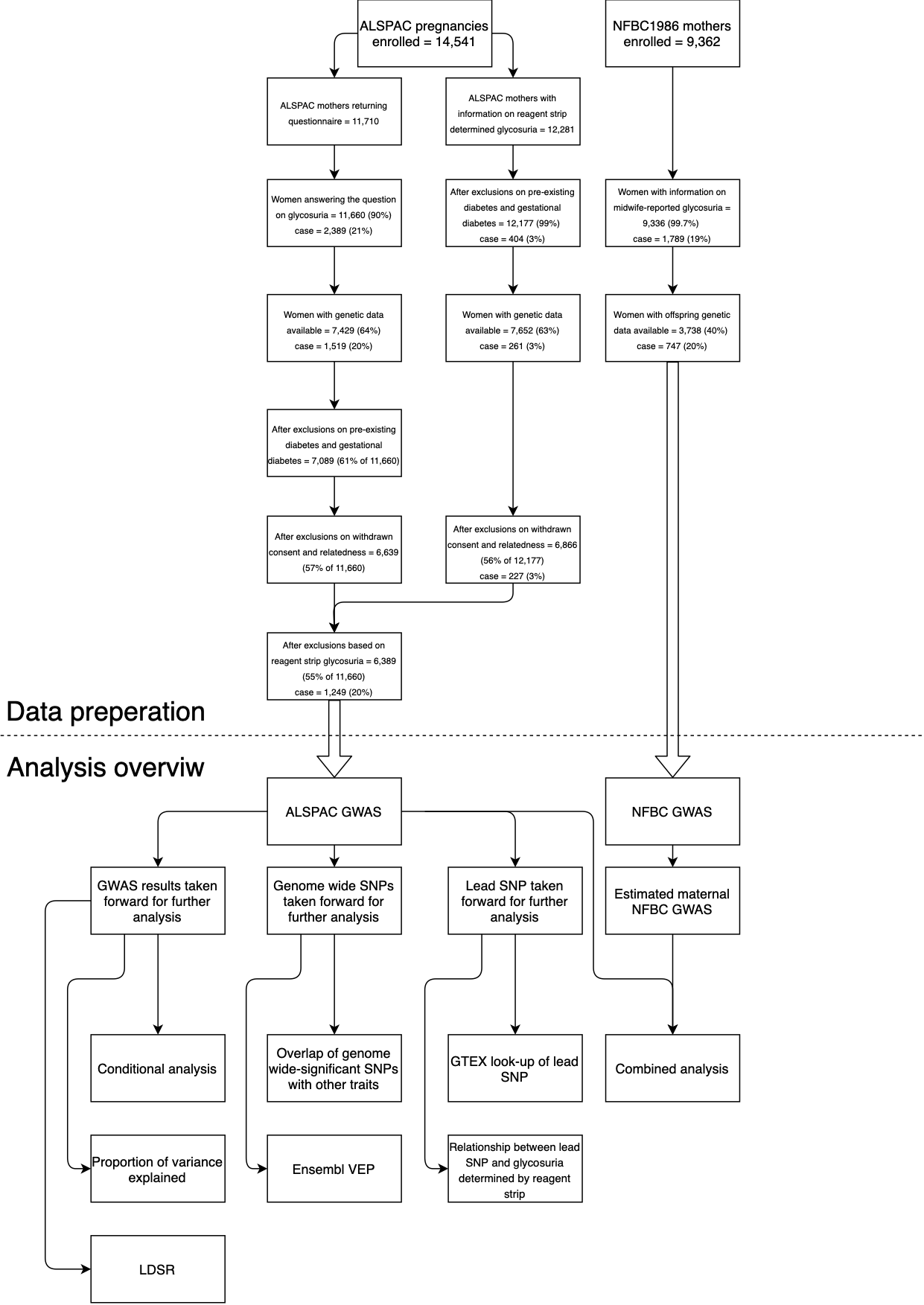

Supplement: Supplementary_Figure_1_ddaa054 [file supplementary_figure_1_ddaa054.png]

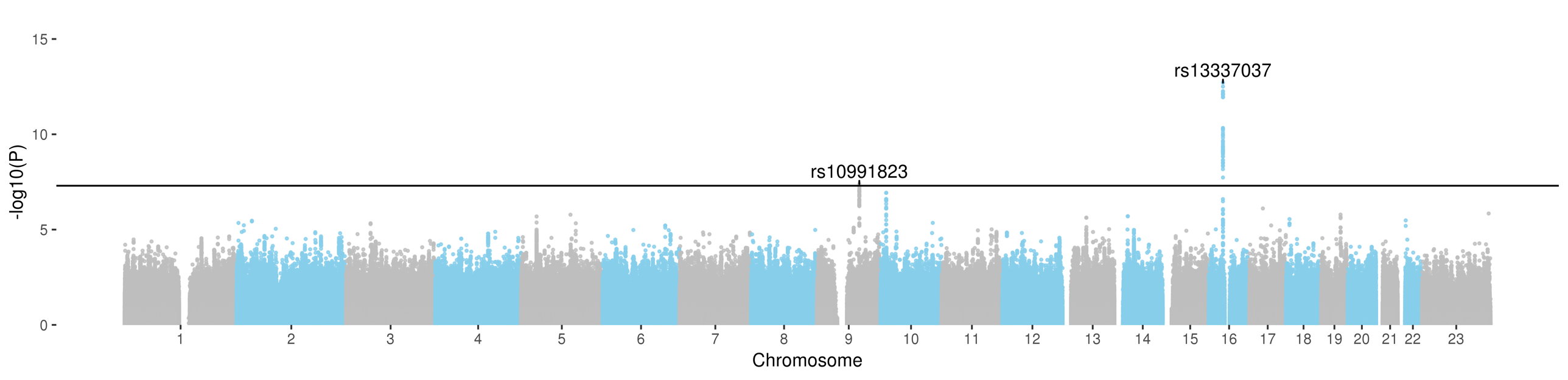

Supplement: Supplementary_Figure_2_ddaa054 [file supplementary_figure_2_ddaa054.png]

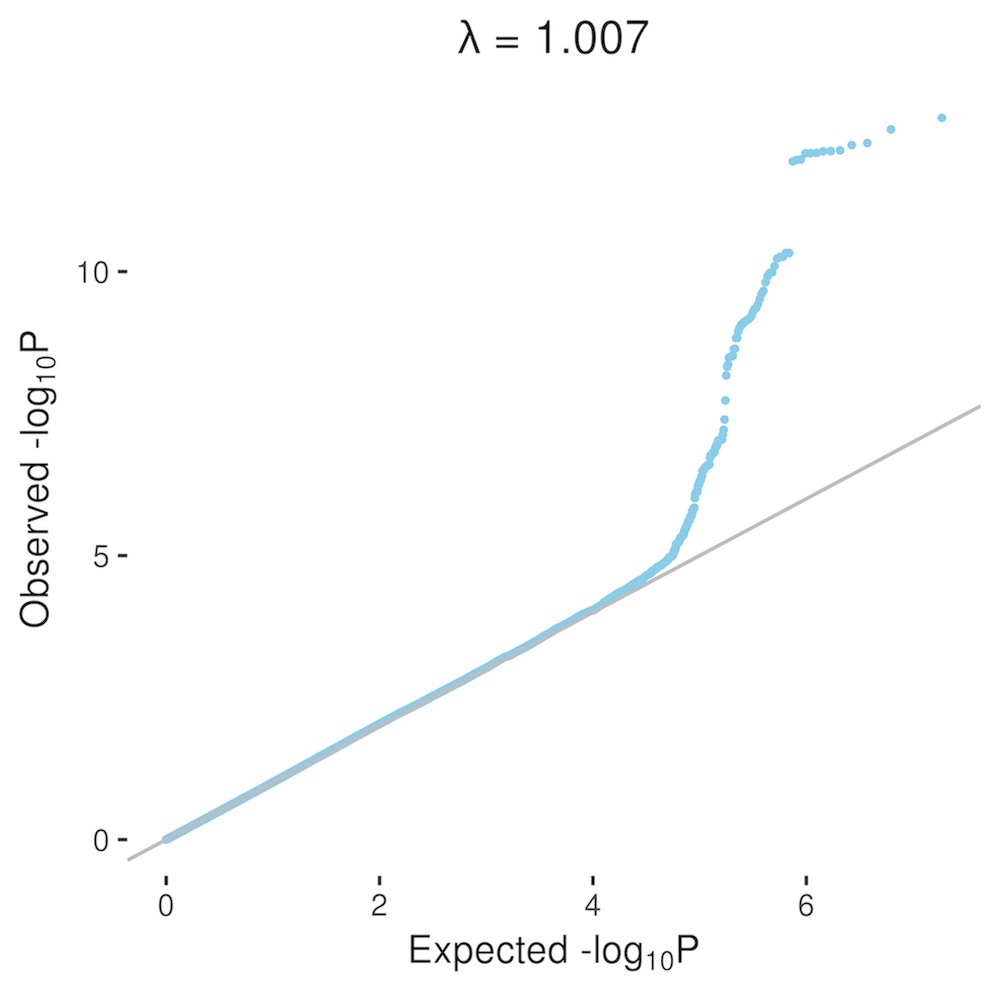

Supplement: Supplementary_Figure_3_ddaa054 [file supplementary_figure_3_ddaa054.jpeg]

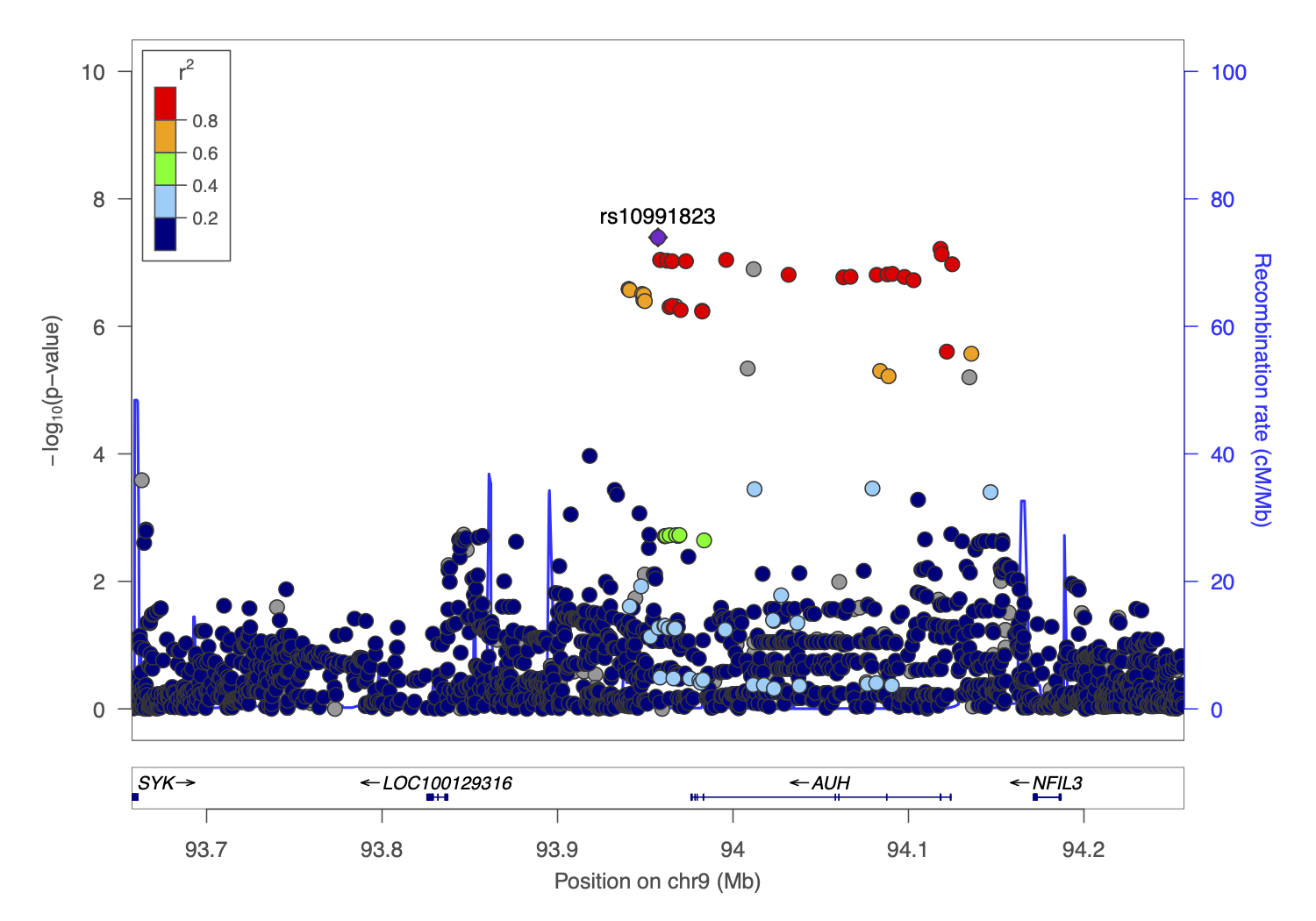

Supplement: Supplementary_Figure_4_ddaa054 [file supplementary_figure_4_ddaa054.png]

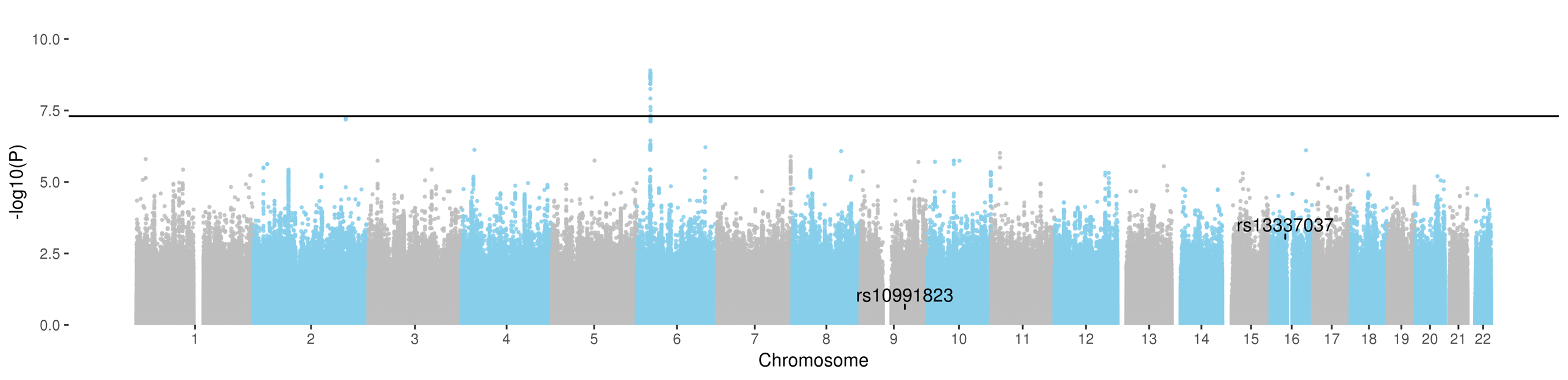

Supplement: Supplementary_Figure_5_ddaa054 [file supplementary_figure_5_ddaa054.png]

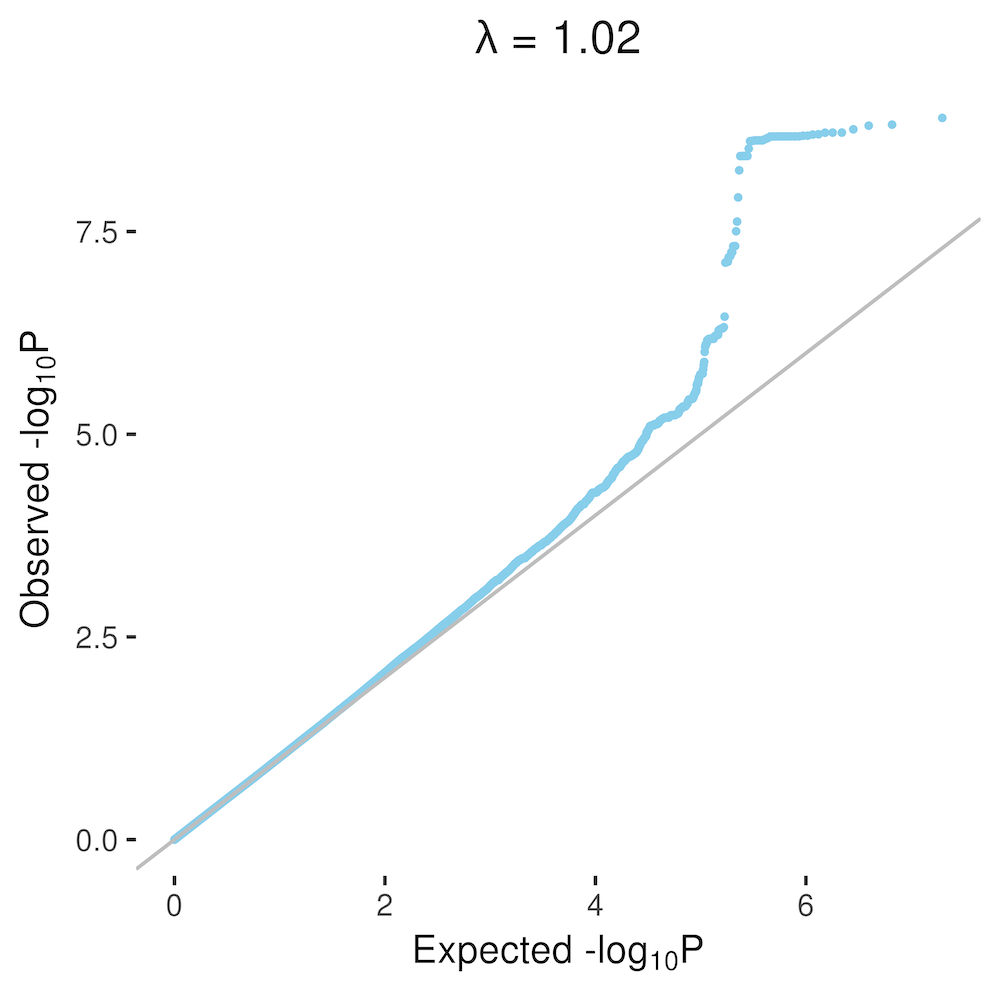

Supplement: Supplementary_Figure_6_ddaa054 [file supplementary_figure_6_ddaa054.png]

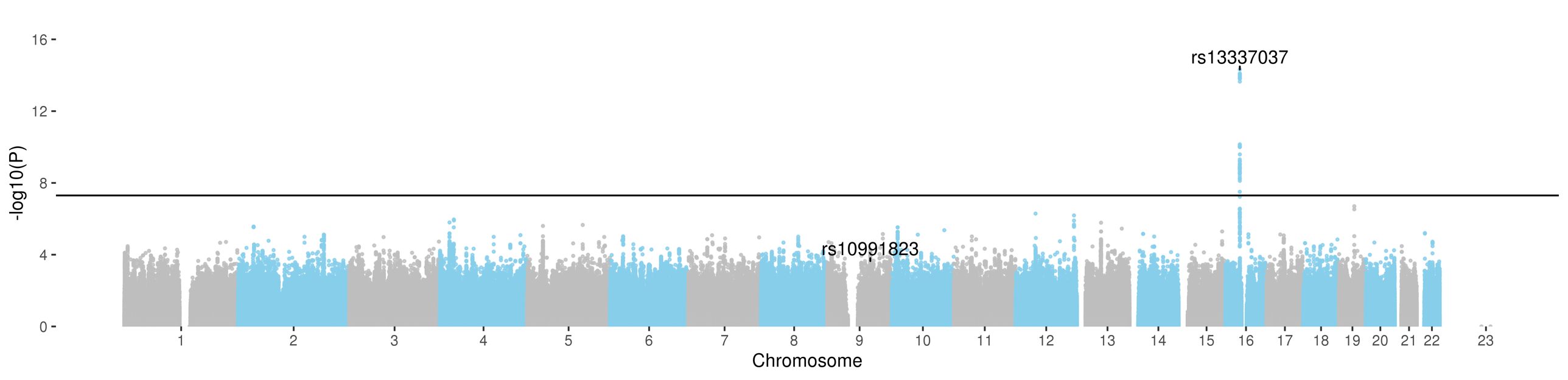

Supplement: Supplementary_Figure_7_ddaa054 [file supplementary_figure_7_ddaa054.png]

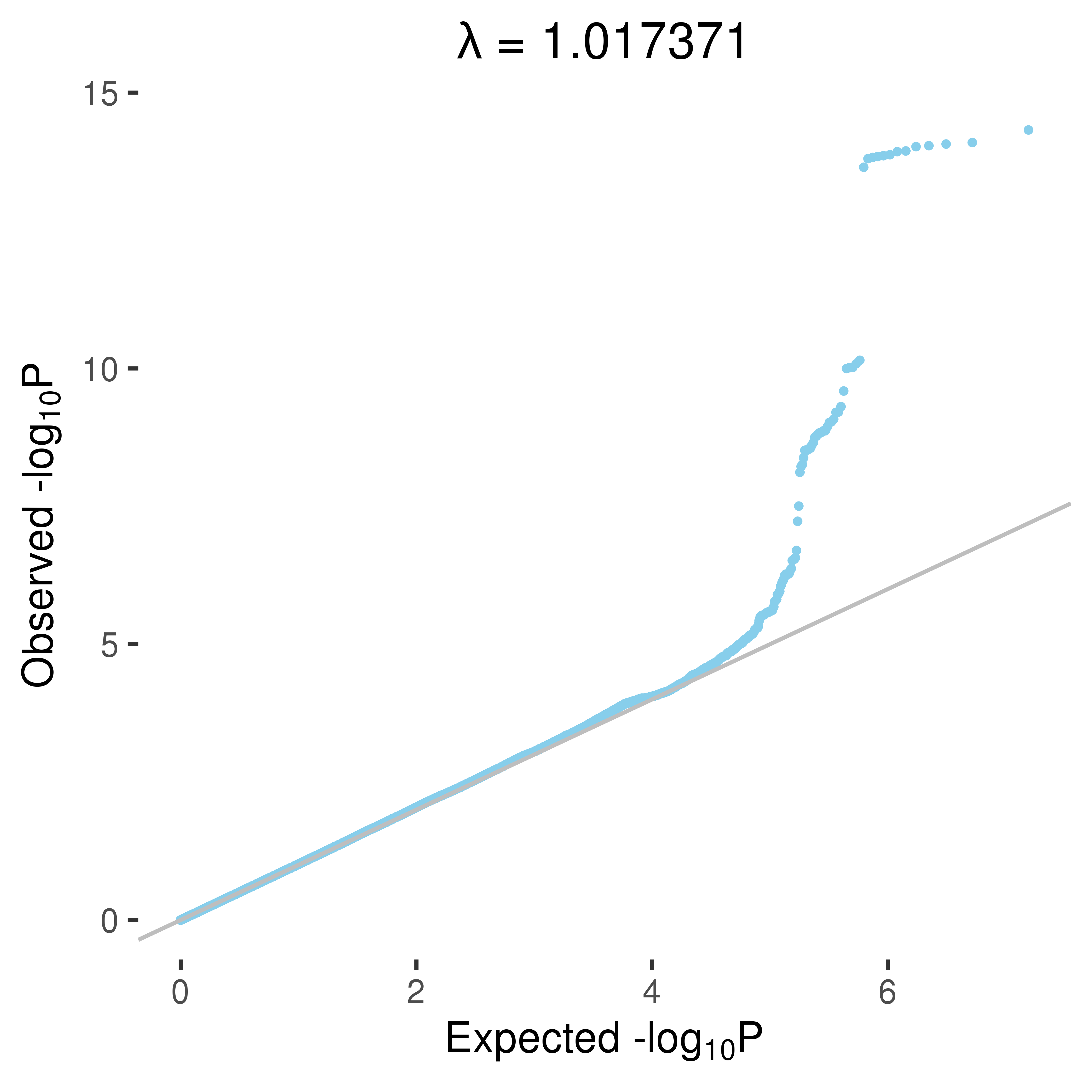

Supplement: Supplementary_Figure_8_ddaa054 [file supplementary_figure_8_ddaa054.png]

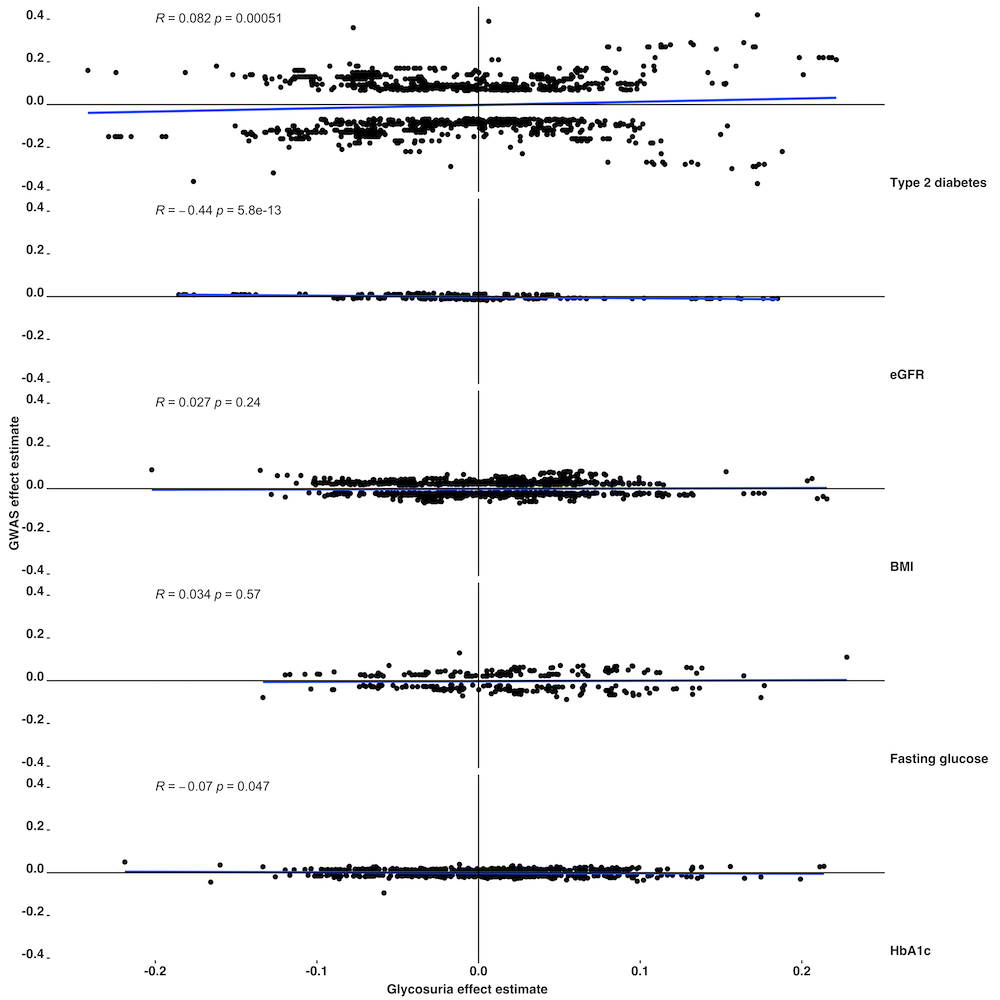

Supplement: Supplementary_Figure_9_ddaa054 [file supplementary_figure_9_ddaa054.png]
