# Supplementary material for: Common variation at 16p11.2 is associated with glycosuria in pregnancy: findings from a genome-wide association study in European women
Source: Hum Mol Genet. 2020 Mar 30;29(12):2098–106. doi: 10.1093/hmg/ddaa054 (PMC7390941; doi:10.1093/hmg/ddaa054)
Supplement: Supplementary_legends_ddaa054 [file supplementary_legends_ddaa054.docx]

**Legends to Supplementary Figures**

**Supplementary Figure 1. Overview of data preparation and analysis.**

The flowchart is an extended version of Figure 1 which includes the data preparation steps performed for the ALSPAC and NFBC data prior to performing genome-wide association studies and subsequent analysis.

**Supplementary Figure 2. Manhattan plot of a GWAS of self-reported glycosuria in the third trimester of pregnancy in ALSPAC**

The line indicates a P value of 5 x 10^-8^. The lead SNPs on chromosomes 9 and 16 are labelled

**Supplementary Figure 3. QQ plot of GWAS of self-reported glycosuria in the third trimester in ALSPAC**

QQ plot of test statistics shows inflation of associations at lower P values. The grey line indicates y=x. The genomic inflation factor (λ) is shown.

**Supplementary Figure 4. Regional association plot of lead association on chromosome 9 (rs10991823) in ALSPAC GWAS.**

P-values (on a −log10 scale) in ALSPAC are shown. Each SNP is coloured according to the degree of linkage disequilibrium (LD) with the lead SNP rs10991823 (shown as a purple diamond). LD values are from the 1000 Genomes (March 2012 release) European population (created using locuszoom.org build: hg19).

**Supplementary Figure 5. Manhattan plot of a GWAS of midwife reported glycosuria in NFBC1986**

The line indicates a P value of 5 x 10^-8^. The lead SNPs on chromosomes 9 and 16 from the ALSPAC GWAS are labelled

**Supplementary Figure 6. QQ plot of GWAS of midwife reported glycosuria in NFBC1986**

QQ plot of test statistics shows inflation of associations at lower P values. The grey line indicates y=x. The genomic inflation factor (λ) is shown.

**Supplementary Figure 7. Manhattan plot of the combined ALSPAC and NFBC1986 GWAS results**

The line indicates a P value of 5 x 10^-8^. The lead SNPs on chromosomes 9 and 16 from the ALSPAC GWAS are labelled

**Supplementary Figure 8. QQ plot of the combined ALSPAC and NFBC1986 GWAS results**

QQ plot of test statistics shows inflation of associations at lower P values. The grey line indicates y=x. The genomic inflation factor (λ) is shown.

**Supplementary Figure 9. Direction of effect estimates from different GWAS' and effect estimates from ALSPAC GWAS**

Scatter plot of effect estimates of associations from different GWAS’ against effect estimates in a GWAS of glycosuria in ALSPAC. The blue line represents the Pearson correlation co-efficient. Effect estimates are the raw effects obtained from the GWAS of each trait; SNPs included are all those whose association in the respective GWAS reached genome wide significance (p-value ≤ 5 x 10^-8^); eGFR: estimated glomerular filtration rate. The Pearson correlation coefficient and associated p-value is shown.

**Legends to Supplementary Tables**

**Supplementary Table 1. SNPs reaching genome-wide significance (P < 5 x 10^-8^) for self-reported glycosuria in ALSPAC mothers**

Results are ordered by P value. rsID: reported rsID; chr: chromosome; position: base position n; EA: effect allele; NEA: non-effect allele/other allele; EAF: effect allele frequency; P: reported p-value for EA; B: effect estimate; SE: standard error of the effect estimate; OR: odds ratio of the effect estimate; CI_lower: lower 95% confidence interval for the OR; CI_upper: upper 95% confidence interval for the OR; info: information score.

**Supplementary Table 2. Number of individuals with self-report glycosuria and reagent strip glycosuria, and Pearson correlation coefficient of the two measures of glycosuria**

PCC gives the Pearson correlation coefficient and associated P value of self-reported glycosuria and reagent strip glycosuria in ALSPAC mothers with available genetic data.

**Supplementary Table 3. SNPs reaching genome-wide significance (P < 5 x 10^-8^) for midwife reported glycosuria in NFBC1986 mothers**

Results are ordered by P value. rsID: reported rsID; chr: chromosome; position: base position n; EA: effect allele; NEA: non-effect allele/other allele; EAF: effect allele frequency; P: reported p value for EA; B: effect estimate; SE: standard error of the effect estimate; OR: odds ratio of the effect estimate; CI_lower: lower 95% confidence interval for the OR; CI_upper: upper 95% confidence interval for the OR; info: information score.

**Supplementary Table 4. SNPs reaching genome-wide significance (P < 5 x 10^-8^) in the combined GWAS results from ALSPAC and NFBC1986 mothers for self-reported and midwife reported glycosuria, respectively**

Results are ordered by P value. rsID: reported rsID; chr: chromosome; position: base position; EA: effect allele; NEA: non-effect allele/other allele; EAF: weighted average of frequency for allele 1 across all studies; P: reported p value for EA; Zscore: the combined z-statistic for this marker; Weight: the sum of the individual study weights calculated as 4/(1/cases + 1/controls); direction in ALSPAC and NFBC of the effect estimate respectively.

**Supplementary Table 5. Validation of the doubling of offspring effect estimates in ALSPAC using logistic regression.**

Trait: the glycosuria phenotype; Group: whether analysis was performed for offspring or maternal genotype; Note: additional information about the effect estimates present, doubled = the effect estimate and standard error were doubled; n: number of controls (i.e., those reporting no glycosuria); n_cases: number of cases (i.e., those reporting yes glycosuria); b = the logistic regression effect estimate; se: standard error of b; OR: odds ratio of the effect estimate; CI.lower/upper: OR lower and upper 95% confidence interval; P: p-value.

**Supplementary Table 6. Overlap of SNPs reaching genome-wide significance in ALSPAC mothers with GWASs for HbA1c, fasting glucose, fasting insulin, type 2 diabetes, BMI, and estimated glomerular filtration rate**

Where NA is present the SNP from the ALSPAC study was not found within the investigated GWAS. SNPs are ordered by lowest P value in the ALSPAC study, with the lead SNP on the first row. rsID: reported rsID; chr: chromosome; position: base position; EA: effect allele; NEA/OA: non-effect allele/other allele; EAF/MAF: effect allele frequency/minor allele frequency (the EAF/MAF was not available in the type 2 diabetes GWAS results obtained from DIAGRAM); B: effect estimate; SE: standard error of the effect estimate; P: reported p value for the EA. MAGIC: Meta-Analyses of Glucose and Insulin-related traits Consortium; DIAGRAM: DIAbetes Genetics Replication And Meta-analysis consortium; GIANT: Genetic Investigation of ANtrhopometric Traits consortium; T2D: type 2 diabetes; BMI: body mass index.

**Supplementary Table 7. Genetic correlation of 832 traits in LD Hub with GWAS of self-reported glycosuria in ALSPAC mothers**

Analysis was performed on 19/02/2019 with all 832 available traits in LD Hub. Data is presented for all traits and is ordered with the 43 pertinent traits first (in order of p-value) followed by all other traits (ordered by smallest to largest p-value). Where NA is present LD score regression analysis was not possible. Trait: the trait for which LD score regression with our GWAS of self-reported glycosuria in ALSPAC mothers was performed; PMID: PubMed identification number of the study the trait is from; Category: category trait falls within as defined by LD Hub; ethnicity: ethnicity of individuals tested in the PMID study for the trait; note: note from LD Hub; rg: LD score regression genetic correlation estimate; SE: standard error of the rg; S: Z statistic; P: P value for the rg.

**Supplementary Table 8. Ensembl Variant Effect Predictor output for the lead SNP on chromosome 16 (rs13337037) and SNPs in high LD (R2 >= 0.8)**

We searched the lead SNP (rs13337037) with both rsID and chromosome:position:EA:NEA (16_31478711_A/G). The lead SNP identified in the ALSPAC GWAS is in bold at the top of the table. Uploaded variation: the input to Ensembl VEP either rsID or chromosome:position:EA; Location: in standard coordinate format (chr:start or chr:start-end); Allele: the variant allele used to calculate the consequence; Consequence: consequence type of this variant; Impact: the impact modifier for the consequence type; Symbol: the gene symbol; Gene: Ensembl stable ID of affected gene; Feature type: type of feature, currently one of Transcript, RegulatoryFeature, MotifFeature; Feature: Ensembl stable ID of feature; Biotype: Biotype of transcript or regulatory feature.

**Supplementary Table 9. GTEx output for the lead SNP on chromosome 16 (rs13337037)**

The table is order by P value. Gencode Id: Gencode identifier; Gene Symbol: gene symbol for Gencode Id; Variant Id: variant identifier of the searcher SNP Id in chromosome_position_NEA_EA format using build 37; SNP Id: searched rsID in GTEx; P: P value of eQTL; NES: normalized effect size; Tissue: tissue in which the rsID is associated with an eQTL.
