# Supplementary material for: Common variation at 16p11.2 is associated with glycosuria in pregnancy: findings from a genome-wide association study in European women
Source: Hum Mol Genet. 2020 Mar 30;29(12):2098–106. doi: 10.1093/hmg/ddaa054 (PMC7390941; doi:10.1093/hmg/ddaa054)
Supplement: HMG-2019-TF-00473_R1_Supplement_LEE_ddaa054 [file hmg-2019-tf-00473_r1_supplement_lee_ddaa054.docx]

**Supplementary Information**

**ALSPAC overview**

Pregnant women resident in Avon, UK with expected dates of delivery 1st April 1991 to 31st December 1992 were invited to take part in the study. The initial number of pregnancies enrolled is 14,541 (for these at least one questionnaire has been returned or a “Children in Focus” clinic had been attended by 19/07/99). Of these initial pregnancies, there was a total of 14,676 foetuses, resulting in 14,062 live births and 13,988 children who were alive at 1 year of age. When the oldest children were approximately 7 years of age, an attempt was made to bolster the initial sample with eligible cases who had failed to join the study originally. As a result, when considering variables collected from the age of seven onwards (and potentially abstracted from obstetric notes) there are data available for more than the 14,541 pregnancies mentioned above. The number of new pregnancies not in the initial sample (known as Phase I enrolment) that are currently represented on the built files and reflecting enrolment status at the age of 24 is 904 (452, 254 and 198 recruited during Phases II, III and IV respectively), resulting in an additional 811 children being enrolled. The phases of enrolment are described in more detail in the cohort profile paper (see footnote 4 below). Please note that phase 4 enrolment (age 18-24) is not currently included in the cohort profile. The total sample size for analyses using any data collected after the age of seven is therefore 15,247 pregnancies, resulting in 15,458 foetuses. Of this total sample of 15,656 foetuses, 14,973 were live births and 14,899 were alive at 1 year of age. A 10% sample of the ALSPAC cohort, known as the Children in Focus (CiF) group, attended clinics at the University of Bristol at various time intervals between 4 to 61 months of age. The CiF group were chosen at random from the last 6 months of ALSPAC births (1432 families attended at least one clinic). Excluded were those mothers who had moved out of the area or were lost to follow-up and those partaking in another study of infant development in Avon.

**ALSPAC genotype data**

ALSPAC children were genotyped using the Illumina HumanHap550 quad chip genotyping platforms by 23andme subcontracting the Wellcome Trust Sanger Institute, Cambridge, UK and the Laboratory Corporation of America, Burlington, NC, US. The resulting raw genome-wide data were subjected to standard quality control methods. Individuals were excluded on the basis of gender mismatches; minimal or excessive heterozygosity; disproportionate levels of individual missingness (>3%) and insufficient sample replication (IBD < 0.8). Population stratification was assessed by multidimensional scaling analysis and compared with Hapmap II (release 22) European descent (CEU), Han Chinese, Japanese and Yoruba reference populations; all individuals with non-European ancestry were removed. SNPs with a minor allele frequency of < 1%, a call rate of < 95% or evidence for violations of Hardy-Weinberg equilibrium (P < 5 x 10^-7^) were removed. Cryptic relatedness was measured as proportion of identity by descent (IBD > 0.1). Related subjects that passed all other quality control thresholds were retained during subsequent phasing and imputation. 9,115 subjects and 500,527 SNPs passed these quality control filters.

ALSPAC mothers were genotyped using the Illumina human660W-quad array at Centre National de Génotypage (CNG) and genotypes were called with Illumina GenomeStudio. PLINK (v1.07) was used to carry out quality control measures on an initial set of 10,015 subjects and 557,124 directly genotyped SNPs. SNPs were removed if they displayed more than 5% missingness or a Hardy-Weinberg equilibrium P value of less than 1.0 x 10^-06^. Additionally, SNPs with a minor allele frequency of less than 1% were removed. Samples were excluded if they displayed more than 5% missingness, had indeterminate X chromosome heterozygosity or extreme autosomal heterozygosity. Samples showing evidence of population stratification were identified by multidimensional scaling of genome-wide identity by state pairwise distances using the four HapMap populations as a reference and then excluded. Cryptic relatedness was assessed using an IBD estimate of more than 0.125 which is expected to correspond to roughly 12.5% alleles shared IBD or a relatedness at the first cousin level. Related subjects that passed all other quality control thresholds were retained during subsequent phasing and imputation. 9,048 subjects and 526,688 SNPs passed these quality control filters.

We combined 477,482 SNP genotypes in common between the sample of mothers and sample of children. We removed SNPs with genotype missingness above 1% due to poor quality (11,396 SNPs removed) and removed a further 321 subjects due to potential ID mismatches. This resulted in a dataset of 17,842 subjects containing 6,305 duos and 465,740 SNPs (112 were removed during liftover and 234 were out of HWE after combination). We estimated haplotypes using ShapeIT (v2.r644) which utilises relatedness during phasing. We obtained a phased version of the 1000 genomes reference panel (Phase 1, Version 3) from the Impute2 reference data repository (phased using ShapeIT v2.r644, haplotype release date Dec 2013). Imputation of the target data was performed using Impute V2.2.2 against the reference panel (all polymorphic SNPs excluding singletons), using all 2186 reference haplotypes (including non-Europeans).

This gave 8,237 eligible children and 8,196 eligible mothers with available genotype data after exclusion of related subjects using cryptic relatedness measures described previously.

**ALSPAC GWAS**

Prior to genome-wide analysis, SNPs were filtered based on an info score threshold of ≥ 0.3 and a minor allele frequency (MAF) threshold of ≥ 0.01. Assuming an additive genetic model, we carried out a GWAS of imputed data on self-reported glycosuria in the third trimester of pregnancy (1,249/5,390 cases/controls) using logistic regression and adjusting for the top ten principal components of genetic ancestry to control for potential confounding by population stratification in SNPTESTv2.5.2, with the options *-frequentist 1* and *-method expected*. Positive responses were coded as the case group and logistic regression at each SNP was carried out with the top ten principal components of genetic ancestry as covariates. Principal components were generated using unrelated individuals (IBS < 0.05) and independent SNPs with long range LD regions removed in PLINK (V1.90) using the *--pca* command.

**ALSPAC offspring analysis**

To investigate whether doubling of effect estimates and standard errors in the NFBC1986 GWAS were appropriate, we investigated the effects of ALSPAC offspring genotype on ALSPAC mothers self-reported glycosuria. We obtained the dosage information for rs13337037 for all mothers and children in ALSPAC. For the mothers, we performed all of the exclusion criteria as with the GWAS, excluding those with withdrawn consent for genotype and phenotype data, and those with pre-existing conditions. We merged the two dosage files, retaining data for all mother-child pairs (n = 5076). Information on self-reported glycosuria and glycosuria determined by reagent strip was available for 4800 (922/3878; case/control) and 5076 (4911/165) individuals, respectively. The number of individuals included is less than the GWAS and previous analysis for glycosuria determined by reagent strip because of exclusions based on the availability of child genotype data .

We performed logistic regressions of rs13337037 dosage genotype in the mothers’ offspring with mothers’ self-reported glycosuria. We performed the same analysis using glycosuria determined by reagent strip. We also performed logistic regression of mother’s genotype and self-reported glycosuria with and without conditioning on offspring genotype. These analyses were performed in R(1) (version 3.6.2).

Logistic regression (Supplementary Table 5) showed that, when doubled, the effect estimate obtained from the analysis of offspring genotype (OR per A allele of self-reported glycosuria: 2.00; 95% CI: 1.60 – 2.50; p-value = 7.4 x 10^-10^) was slightly higher than (though statistically consistent with) that derived from the equivalent analysis performed using mother’s own genotype (OR per A allele of self-reported glycosuria: 1.45; 95% CI 1.30 – 1.61; p-value = 3.54 x 10^-11^). One possible explanation for a higher point estimate could be that offspring genotype has a direct effect on the maternal phenotype (i.e., on the mother’s risk of developing glycosuria during pregnancy), however it is difficult to generate precise estimates of this given sample size for this analysis. Additionally, results of a logistic regression of maternal genotype and self-reported glycosuria conditional on offspring genotype (OR per A allele of self-reported glycosuria: 1.71; 95% CI: 1.16 – 2.53; p-value = 0.007) did not provide compelling evidence of child specific effects.

**Overlap of genome-wide significant SNPs in ALSPAC with other GWASs**

List of GWAS summary level data used to investigate the overlap between SNPs identified in ALSPAC and other GWASs.

- Meta-Analyses of Glucose and Insulin-related traits Consortium (MAGIC; [www.magicinvestigators.org](http://www.magicinvestigators.org), date accessed 08/01/2019)
  - HBA1c in up to 123,665 Europeans(2) (<ftp://ftp.sanger.ac.uk/pub/magic/HbA1c_METAL_European.txt.gz>)
  - Fasting insulin in up to 46,186 Europeans(3) (<ftp://ftp.sanger.ac.uk/pub/magic/MAGIC_ln_FastingInsulin.txt>)
  - Fasting glucose in up to 46,186 Europeans(3) (ftp://ftp.sanger.ac.uk/pub/magic/MAGIC_FastingGlucose.txt)
- DIAbetes Genetics Replication And Meta-analysis consortium (DIAGRAM; <http://diagram-consortium.org/>, date accessed 08/01/2019)
  - Type 2 diabetes in up to 26,676 cases and 132,532 controls Europeans(4) (<http://diagram-consortium.org/downloads/DIAGRAM_1000G_GWAS.pdf>)
- Genetic Investigation of ANtrhopometric Traits consortium (GIANT; https://portals.broadinstitute.org/collaboration/giant/index.php/GIANT_consortium_data_files, date accessed 08/01/2019)
  - Body mass index in up to 322,154 Europeans(5) (<https://portals.broadinstitute.org/collaboration/giant/images/1/15/SNP_gwas_mc_merge_nogc.tbl.uniq.gz>, date accessed 08/01/2019).
- GWAS Catalog (<https://www.ebi.ac.uk/gwas/home>)
  - Estimated glomerular filtration rate (eGFR) in up to 110,517 Europeans(6) (accession number = GCST004292; <ftp://ftp.ebi.ac.uk/pub/databases/gwas/summary_statistics/GorskiM_28452372_GCST004292>; date accessed 19/02/19)

References

1 Alto, W.A. (2005) No need for routine glycosuria/proteinuria screen in pregnant women. *J. Fam. Pract.*, **54**, 978-983.

2 Wheeler, E., Leong, A., Liu, C.-T., Hivert, M.-F., Strawbridge, R.J., Podmore, C., Li, M., Yao, J., Sim, X., Hong, J. *et al.* (2017) Impact of common genetic determinants of Hemoglobin A1c on type 2 diabetes risk and diagnosis in ancestrally diverse populations: A transethnic genome-wide meta-analysis. *PLoS medicine*, **14**, e1002383-e1002383.

3 Dupuis, J., Langenberg, C., Prokopenko, I., Saxena, R., Soranzo, N., Jackson, A.U., Wheeler, E., Glazer, N.L., Bouatia-Naji, N., Gloyn, A.L. *et al.* (2010) New genetic loci implicated in fasting glucose homeostasis and their impact on type 2 diabetes risk. *Nature genetics*, **42**, 105-116.

4 Scott, R.A., Scott, L.J., Mägi, R., Marullo, L., Gaulton, K.J., Kaakinen, M., Pervjakova, N., Pers, T.H., Johnson, A.D., Eicher, J.D. *et al.* (2017) An Expanded Genome-Wide Association Study of Type 2 Diabetes in Europeans. *Diabetes*, **66**, 2888-2902.

5 Locke, A.E., Kahali, B., Berndt, S.I., Justice, A.E., Pers, T.H., Day, F.R., Powell, C., Vedantam, S., Buchkovich, M.L., Yang, J. *et al.* (2015) Genetic studies of body mass index yield new insights for obesity biology. *Nature*, **518**, 197-206.

6 Gorski, M., van der Most, P.J., Teumer, A., Chu, A.Y., Li, M., Mijatovic, V., Nolte, I.M., Cocca, M., Taliun, D., Gomez, F. *et al.* 1000 Genomes-based meta-analysis identifies 10 novel loci for kidney function. in press.
